# Epidemiology, Treatment Patterns, and Cost Analysis of Immune Thrombocytopenia in Spain between 2014 and 2020: A Population-based Study

**DOI:** 10.1055/a-2336-1062

**Published:** 2024-07-08

**Authors:** Tomás J. González-López, Gabriela Alperovich, Elena Burillo, Marta Espejo-Saavedra Soler, Elena Rebollo-Gómez, Ignacio Hernández, Jose L. Justicia, María L. Lozano

**Affiliations:** 1 Department of Haematology, Hospital Universitario de Burgos, Burgos, Spain; 2 Swedish Orphan Biovitrum AB, Madrid, Spain; 3 Atrys Health S.A., Madrid, Spain; 4 Department of Haematology, Hospital General Universitario José María Morales Meseguer, Centro Regional de Hemodonación, Universidad de Murcia, IMIB-Pascual Parrilla, CIBERER-ISCIII, Murcia, Spain

**Keywords:** primary immune thrombocytopenia, adult, paediatric

## Abstract

**Background** Immune thrombocytopenia (ITP) is characterised by low platelet counts and often leads to bleeding, fatigue, and reduced health-related quality of life.

**Methods** This observational, retrospective, population-based study using BIG-PAC® database included Spanish paediatric and adult patients with primary ITP diagnosed in primary care and hospitals between 2014 and 2020 (median follow-up: 4 years). Epidemiology, baseline/clinical characteristics, treatment trends, healthcare resources and costs were analysed.

**Results** The BIG-PAC® database contains records of 1,818,588 patients; 170 adults and 27 children with ITP were included in our analysis. ITP prevalence and annual incidence per 100,000 were estimated in 10.8 (2.8 in chronic ITP [cITP] patients) and 1.5 (0.3 in cITP patients), respectively. Epistaxis was the most common bleeding event, followed by genitourinary and gastrointestinal bleeding; >50%/> 75% of ITP/cITP patients reported fatigue. Chronic patients had lower platelet counts at baseline and required more transfusions. Corticosteroids, immunosuppressants, and thrombopoietin receptor agonists were the most used agents in first-, second- and third-line treatment, respectively. Thirty-five patients, all of them in chronic phase, underwent splenectomy. Patients had on average 13.9, 6.6, and 1.2 visits/year to primary care, haematology/internal medicine, and emergency departments, respectively. More than one-fourth of adult patients took on average 16.3 days of sick leave annually. Mean annual total health care costs were €10,741 (ITP patients) and €19,809 (cITP patients).

**Conclusion** This is the first study to provide an overall perspective on the situation of the Spanish ITP population in terms of epidemiology, treatment trends, health care resources and costs, highlighting unmet patient needs, and direct and indirect costs/resource use between 2014 and 2020.

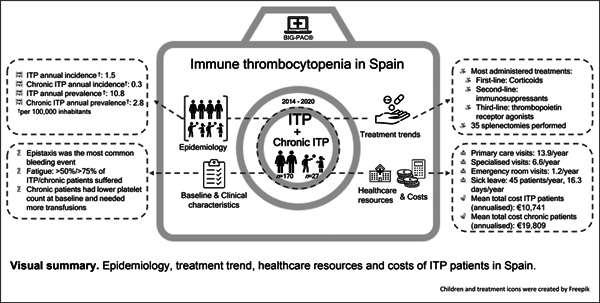

## Introduction

Immune thrombocytopenia (ITP) is an autoimmune haematological disorder caused by a decrease in the number of platelets (<100 × 10 ^9^ /L). 1 2 3 This reduction is thought to result in part from autoantibodies that target platelets or megakaryocytes, which can then impair platelet production, or from T-cell dysregulation. 4 5 Thrombocytopenia may last <3 months (ITP of recent diagnosis), between 3 and 12 months (persistent), or >12 months (chronic), and may cause bleeding, 1 2 6 fatigue, 7 and reduced health-related quality of life (HRQoL). 1 7 8 ITP is classified as primary or secondary, 3 depending on the reduced platelet counts being caused by another disorder or not. 2 3 Children rarely have significant bleeding and their ITP usually resolves spontaneously, unlike adults whose ITP becomes chronic in 80% of cases. 2 4

The annual incidence and the prevalence of primary ITP in adults have been estimated to be 3.3 per 100,000 and 9.5 per 100,000, respectively, 9 but these figures may vary depending on the country analysed. 2 The Orphanet database estimates a prevalence of ITP of 1 to 5 per 10,000 and an annual incidence of 1 in 25,600 to 37,000 in Europe. 10

The treatment focuses on preventing or treating bleeding by increasing patients' platelet counts (>20–30 × 10 ^9^ ), and on improving their HRQoL. 11 Treatment should be individualised for each patient, taking into account the severity of the disease. 11 12 Therapy has evolved over the last decade, and immunosuppressive agents, maintenance treatment with corticosteroids, rituximab or splenectomy, which entail undesirable side effects and have in some cases reduced effectiveness, have given way to other drugs: the second-generation thrombopoietin receptor agonists (TPO-RAs) and, more recently, fostamatinib, while other molecules are still under development. 6 13 14 The current standard of care for newly diagnosed adult patients is corticosteroids (most likely prednisone or dexamethasone) or intravenous immunoglobulin (IVIG) if there is significant bleeding. 15 16 In general, TPO-RAs are the recommended therapy for subsequent lines of treatment over rituximab and splenectomy, always considering the patient's preferences. 16 Most children (80–90%) recover from ITP in <12 months; for those who do not, the choice of treatment is challenging due to the potential side effects of different approaches at such a young age, although the TPO-RAs romiplostim or eltrombopag are the proposed treatment of choice. 17

Characterisation of different variables including treatment of ITP has been studied in other countries, 18 19 20 but no national data are available for Spain. Here, we describe the epidemiological characteristics of patients with ITP and chronic ITP (cITP) treated in Spanish primary care centres and hospitals between 2014 and 2020. With the aim to have an updated and global vision of various aspects related to ITP in Spain, we analysed the patients' baseline and clinical characteristics, their treatment and management, and the health resources and costs derived from their management in primary care and hospitals.

## Materials and Methods

### Study Design and Population

This retrospective observational study was conducted using electronic medical records (EMRs) of patients with ITP diagnosed between January 1, 2014, and December 31, 2020. ITP was defined according to the International Classification of Diseases, 9th Edition, Clinical Modification (ICD-9-CM) codes: 287.31 and 287.39. EMRs were obtained from the BIG-PAC® administrative database (data source: secondary; owner: Atrys Health-RLD), which contains anonymised records of 1,818,588 patients from the Spanish National Health System. These data belong to primary health care centres and referral hospitals from seven health care zones in seven different Spanish autonomous communities and are anonymised prior to inclusion in the BIG-PAC® database to avoid any type of individual identification of the patient or related health care information, such as hospitals or treating physicians. Each patient is assigned a unique code to avoid data duplication. The BIG-PAC® database is a unification of public clinical data from different health care areas. The intermediary company running the software of the clinical centres also unifies fields in the patient's clinical history. Some fields may appear differently, depending on the templates of the Spanish Autonomous Communities (i.e., age may be found in different sections or under different titles of the clinical history in one Autonomous Community or another). Atrys Health does not have access to the primary data source. The BIG-PAC® database has been approved, validated, and registered by the European Medicines Agency (EMA) and data processing is in accordance with the Spanish Personal Data Protection Act.

Patients were divided into paediatric (<18 years old) and adult (≥18 years old) groups for analysis and were followed until death or the end of the study. The index date was the date of the ITP diagnosis. From their diagnosis, patients were followed and classified in patients with recent diagnosis (the last ITP registry would take place within 3 months from diagnosis), persistent (the last ITP registry would take place from 3 to 12 months from diagnosis), and chronic (the last ITP registry would take place >12 months from diagnosis). A subanalysis of the cITP population was performed for some of the variables. Inclusion and exclusion criteria are shown in Supplementary Table S1 (available in the online version only).

### Study Variables

#### Baseline Characteristics

Sociodemographic characteristics of paediatric and adult patients (age and sex) were recorded at the index date, while comorbidities (hypertension, diabetes, alcohol consumption, cardiovascular disease, gastrointestinal disease, infectious complications, bone disease, fractures, thyroid disease, and malignant neoplasms) were recorded in the 6 months prior to the index date. The Charlson comorbidity index (CCI) was calculated to reflect general comorbidity and to approximate the general status of patients. Data on the total ITP population and cITP patients were analysed.

#### Clinical Characteristics

Signs and symptoms of ITP such as bleeding episodes (intracranial, gastrointestinal, genitourinary, nasal bleeding [epistaxis], and bleeding at other sites [specified in Supplementary Table S2 {available in the online version only}]), major bleeding (at any location) requiring hospital admission, fatigue/asthenia, transfusions, or all-cause mortality (if information was available) were estimated during the follow-up period. Major bleeding was defined as any bleeding that could lead to hospitalization. ICD-9-CM codes are shown in Supplementary Table S2 (available in the online version only). Platelet count was measured at baseline and at the end of follow-up. All data were estimated separately for the paediatric and adult populations.

#### Epidemiological Data

Prevalence and incidence were calculated independently of other outcomes. They were estimated using data from 31 December 2021. Prevalence was calculated as the total number of active cases with a diagnosis of ITP in the population analysed in that year, and incidence was calculated as the number of new cases diagnosed per 100,000 patients/year among the patients being actively managed in 2021, without standardisation for patient age/sex (the BIG-PAC® database is similar to the Spanish population pyramid). 21 Data were available for total ITP and cITP patients.

#### Treatment Trends

Treatments received by paediatric and adult patients were analysed at the time of first diagnosis of ITP (12 months prior to the index date) and during the follow-up period of the study using drug-dispensing records. The drugs of interest were IVIG, danazol, cyclophosphamide, rituximab, mycophenolate, cyclosporine, azathioprine, spleen tyrosine kinase inhibitors (fostamatinib), TPO-RAs (romiplostim and eltrombopag), and corticosteroids (dexamethasone, methylprednisolone, prednisolone, prednisone).

They were prescribed according to medical practice; their Anatomical Therapeutic Chemical Classification System codes are given in Supplementary Table S3 (available in the online version only). Splenectomies were analysed during follow-up, but also 2 years before index date to understand changes in treatment patterns (codes in Supplementary Table S3 [available in the online version only]).

### Health Care Resources and Costs

Resource use included those for general practice related to ITP (primary care visits, emergency department visits, hospital admission rate, and length of stay), specialised care visits (haematology and internal medicine), diagnostic tests (laboratory tests [any conventional lab analysis request], conventional radiology, computed tomography [CT], nuclear magnetic resonance [NMR], other tests [lumbar puncture, bone marrow examination and scintigraphy]), and work disability. Direct health care costs were estimated by the frequency with which resources were used during follow-up and their unit cost (based on hospital accounts, Supplementary Table S4 [available in the online version only]). In the case of medical prescriptions, the retail price/package at the time of prescription was obtained from Bot Plus, a database of the General Council of Colleges of Official Pharmacists of Spain. 22 The cost of lost productivity (indirect health care costs) was estimated on the basis of the number of days and percentage of patients on sick leave due to temporary or permanent disability in people aged <65 years old and the mean salary of the Spanish population, according to the National Institute of Statistics. 23

### Statistical Methods

Descriptive univariate statistical analyses were performed for the variables of interest in each study group. Qualitative variables were described using absolute and relative frequencies ( *N* , %), and quantitative variables with means and standard deviations (SDs; symmetric distributions) or medians and interquartile ranges (P25–P75; Q1–Q3; asymmetric distributions). The 95% confidence intervals (CIs) were calculated for the estimation of population parameters.

SPSSWIN version 27 was used for statistical analysis; *p* -values <0.05 were considered statistically significant.

## Results

### Baseline Characteristics

**Fig. 1:**
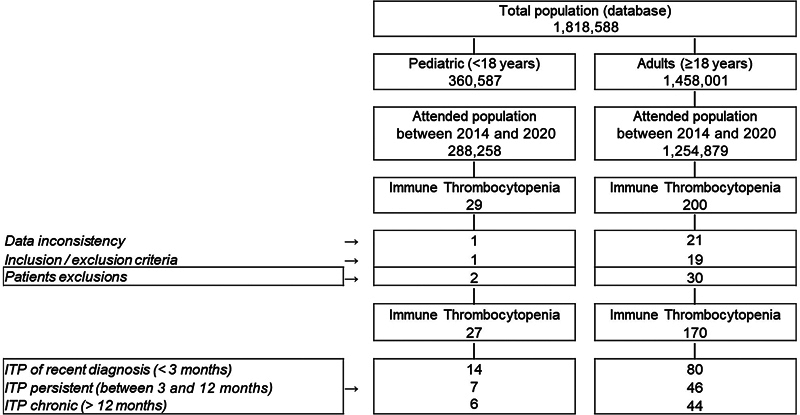
Study overview. ITP, immune thrombocytopenia.

In 2021, the BIG-PAC® database contained information on 1,818,588 patients from public primary care centres and hospitals in Spain. Due to the nature of our analysis, we divided this population into two groups according to their age: paediatric population (<18 years old) and adult population (≥18 years old). This reduced the two groups to 360,587 and 1,458,001 patients, respectively. From 2014 to 2020, 288,258 children and 1,254,879 adults sought medical care. Two patients initially diagnosed with ITP were excluded due to data inconsistency or inclusion/exclusion criteria ( Supplementary Table S1 [available in the online version only]). A final diagnosis of ITP was established in 27 children and 170 adults. Of these, 6 and 44 were diagnosed with cITP, respectively. A summary of attrition is shown in Fig. 1 .

The mean age of the children with cITP was 9.9 years while that of the adults was 69.2 years. Most patients in the cITP subgroups were female (83.3% of children and 59.1% of adults). In both groups, patients with cITP were older than their counterparts in the overall ITP population (mean age was 9.9 [SD 3.4] vs. 6.7 [SD 3.5] years old in children and 69.2 [SD 15.4] vs. 58.5 [SD 18.4] years old in adults). In general, patients with cITP had more comorbidities than the overall ITP group (mean 3.8 [SD 1.9] vs. 2.5 [SD 1.8]). The most common comorbidities in ITP patients were arterial hypertension (25.9%), infectious diseases (12.7%), diabetes (12.2%), and cardiovascular diseases (11.2%). The distribution of these comorbidities in patients with cITP was 46.0, 14.0, 26.0, and 12.0%, respectively. In addition, comorbidities such as previous bleeding (14.0%), anaemia (12.0%), neoplasm (12.0%), or gastrointestinal disease (12.0%) were also common in cITP patients.

The mean CCI was 0.9 [SD 1.3] in the ITP group, and 2.4 [SD 1.6] in the cITP group; most adult patients in the cITP group had a CCI score of ≥2 (88.6%). Children with cITP had a CCI of 0. Baseline characteristics are outlined in Table 1 .

**Table 1:** Baseline characteristics (demographics and comorbidities) of the study population

	Total ITP population				Chronic ITP population			
Study groups	Paediatric (<18 years)	Adults (≥18 years)	Total	*p* -Value	Paediatric (<18 years)	Adults (≥18 years)	Total	*p* -Value
Number of patients	27 (13.7%)	170 (86.3%)	197 (100%)		6 (12.0%)	44 (88.0%)	50 (100%)	
**Sociodemographic characteristics**								
Average age (SD), years	6.7 (3.5)	58.5 (18.4)	51.4 (24.8)	**<0.001**	9.9 (3.4)	69.2 (15.4)	62.1 (24.3)	**<0.001**
Age range (years)								
1–6	17 (63.0)	0	17 (8.6)	**<0.001**	1 (16.7)	0	1 (2.0)	**<0.001**
7–17	10 (37.0)	0	10 (5.1)		5 (83.3)	0	5 (10.0)	
18–44	0	42 (24.7)	42 (21.3)		0	4 (9.1)	4 (8.0)	
45–64	0	61 (35.9)	61 (31.0)		0	13 (29.5)	13 (26.0)	
≥ 65	0	67 (39.4)	67 (34.0)		0	27 (61.4)	27 (54.0)	
Gender (female), *N* (%)	15 (55.6)	94 (55.3)	109 (55.3)	0.980	5 (83.3)	26 (59.1)	31 (62.0)	0.484
**Associated comorbidities, N (%)**								
Arterial hypertension	1 (3.7)	50 (29.4)	51 (25.9)	**0.005**	1 (16.7)	22 (50.0)	23 (46.0)	0.199
Diabetes	1 (3.7)	23 (13.5)	24 (12.2)	0.147	0 (0)	13 (29.5)	13 (26.0)	0.319
Alcoholism	0 (0.0)	6 (3.5)	6 (3.0)	0.321	0 (0)	4 (9.1)	4 (8.0)	0.999
Cardiovascular diseases a	1 (3.7)	21 (12.4)	22 (11.2)	0.185	0 (0)	6 (13.6)	6 (12.0)	0.999
Gastrointestinal diseases	2 (7.4)	14 (8.2)	16 (8.1)	0.884	1 (16.7)	5 (11.4)	6 (12.0)	0.556
Infectious diseases	10 (37.0)	15 (8.8)	25 (12.7)	**<0.001**	1 (16.7)	6 (13.6)	7 (14.0)	0.999
Bone diseases	1 (3.7)	12 (7.1)	13 (6.6)	0.514	0 (0)	3 (6.8)	3 (6.0)	0.999
Fractures	1 (3.7)	7 (4.1)	8 (4.1)	0.919	0 (0)	5 (11.4)	5 (10.0)	0.999
Thyroid disease	1 (3.7)	10 (5.9)	11 (5.6)	0.647	0 (0)	4 (9.1)	4 (8.0)	0.999
Neoplasms (malignant tumours)	1 (3.7)	9 (5.3)	10 (5.1)	0.727	1 (16.7)	5 (11.4)	6 (12.0)	0.556
Previous bleeding	2 (7.4)	12 (7.1)	14 (7.1)	0.948	0 (0)	7 (15.9)	7 (14.0)	0.576
Anaemia	1 (3.7)	9 (5.3)	10 (5.1)	0.727	0 (0)	6 (13.6)	6 (12.0)	0.999
**General comorbidity**								
Chronic diseases, mean (SD)	0.9 (0.9)	2.8 (1.7)	2.5 (1.8)	**<0.001**	1 (1.5)	4.2 (1.6)	3.8 (1.9)	**<0.001**
Charlson index, mean (SD)	0.2 (0.4)	1.0 (1.3)	0.9 (1.3)	**0.002**	0 (0)	2.7 (1.4)	2.4 (1.6)	**<0.001**
Charlson index, *N* (%)								
0	22 (81.5)	80 (47.1)	102 (51.8)	**0.010**	6 (100)	0 (0)	6 (12.0)	**<0.001**
1	5 (18.5)	46 (27.1)	51 (25.9)		0 (0)	5 (11.4)	5 (10.0)	
≥2	0 (0.0)	44 (25.9)	44 (22.3)		0 (0)	39 (88.6)	39 (78.0)	

Abbreviation: ITP, immune thrombocytopenia; SD, standard deviation.

Values are expressed in *N* (percentage) or mean (SD). *p* -Value denotes statistical significance.

a Included: Ischemic cardiopathy, stroke, and heart failure.

### Clinical Characteristics

Clinical characteristics are shown in Table 2 . Mean and median follow-up in the overall ITP population were very similar between paediatric and adult patients. Patients were followed up for a mean/median of 4 years; 47.7% were newly diagnosed. The most common bleeding event in both children and adults was epistaxis (61.9%), followed by genitourinary bleeding (15.2%), and gastrointestinal bleeding (7.6%). Bleeding at other sites represented the 21.8% of cases. Intracranial bleeding was reported in only one adult patient ( Table 2 ). Major bleeding requiring hospital admission was 8.1%.

**Table 2:** Clinical variables associated with immune thrombocytopenic

	Total ITP population				Chronic ITP population			
Study groups	Paediatric (<18 years)	Adults (≥18 years)	Total	*p-* Value	Paediatric (<18 years)	Adults (≥18 years)	Total	*p* -Value
Number of patients	27 (13.7%)	170 (86.3%)	197 (100%)		6 (12%)	44 (88%)	50 (100%)	
**Follow-up period, years**								
Mean (SD)	4.1 (1.2)	4.1 (1.8)	4.1 (1.7)	0.587	4.4 (1.9)	3.3 (1.9)	3.4 (1.9)	0.210
Median (P25; P75)	4 (4–5)	4 (3–6)	4 (3–6)		4.4 (4.2–5.4)	3.4 (1.5–4.8)	3.6 (1.6–5)	
**Follow-up period, patients (ITP),** ***N*** **(%)**								
ITP of recent diagnosis a	14 (51.9)	80 (47.1)	94 (47.7)	0.884				
Persistent ITP b	7 (25.9)	46 (27.1)	53 (26.9)					
Chronic ITP c	6 (22.2)	44 (25.9)	50 (25.4)					
**Bleeding events,** ***N*** **(%)**								
Intracranial bleeding	0 (0.0)	1 (0.6)	1 (0.5)	0.689	0 (0)	1 (2.3)	1 (2.0)	0.999
Gastrointestinal bleeding	1 (3.7)	14 (8.2)	15 (7.6)	0.410	1 (16.7)	9 (20.5)	10 (20.0)	0.999
Genitourinary bleeding	3 (11.1)	27 (15.9)	30 (15.2)	0.522	0 (0)	8 (18.2)	8 (16.0)	0.572
Other events	9 (33.3)	34 (20.0)	43 (21.8)	0.119	0 (0)	10 (22.7)	10 (20.0)	0.327
Nasal bleeding (epistaxis)	15 (55.6)	107 (62.9)	122 (61.9)	0.463	2 (33.3)	37 (84.1)	39 (78.0)	**0.017**
Major bleeding with hospital admission	2 (7.4)	14 (8.2)	16 (8.1)	0.884	1 (16.7)	7 (15.9)	8 (16.0)	0.999
**Other variables,** ***N*** **(%)**								
Fatigue/Asthenia	13 (48.1)	96 (56.5)	109 (55.3)	0.419	4 (66.7)	34 (77.3)	38 (76.0)	0.621
Transfusions	2 (7.4)	15 (8.8)	17 (8.6)	0.808	1 (16.7)	8 (18.2)	9 (18.0)	0.999
Death (all causes)	0 (0)	2 (1.2)	2 (1.0)	0.571	0 (0)	1 (2.3)	1 (2.0)	0.999
**Clinical parameters**								
* Platelet count: initial (baseline), × 10 ^9^ /L *								
Mean (SD)	12.5 (8.5)	13.9 (7.8)	13.7 (7.8)	0.658	10.5 (9)	12.4 (7)	12.2 (7)	0.386
Median (P25; P75)	11 (5–18)	13 (8–18)	12 (8–18)		8 (4.5–12)	10 (7–18)	10 (7–18)	–
* Platelet count, end of follow-up, × 10 ^9^ /L *								
Mean (SD)	127.8 (23.6)	125.7 (19.6)	126 (20.2)	0.692	123 (32)	122.3 (21)	122.4 (22)	0.581
Median (P25; P75)	121 (110–145)	122 (112–135)	122 (112–137)		106 (103.25–136)	116 (105–135)	115 (104.2–135)	–

Abbreviation: ITP, immune thrombocytopenia; P, percentile; SD, standard deviation.

Values are expressed in *N* (percentage) or mean (SD). *p* -Value denotes statistical significance.

a Three months from diagnosis.

b Between 3 and 12 months from diagnosis.

c More than 12 months from diagnosis.

In patients with cITP, median follow-up was 3.6 (1.6–5) years; children had longer follow-up than adults, although this was not statistically significant. The most common bleeding events in this population were epistaxis (which was less frequent in children [33.3%] than adults [84.1%]), and gastrointestinal bleeding (20.0%). Genitourinary (18.2%) and intracranial (2.3%) bleeding were reported only in the adult population, as well as bleeding at other sites (22.7%). Major bleeding requiring hospitalisation was present in 16.0% of the population ( Table 2 ).

Regarding other variables, more than half of the patients in the ITP group and >75% of the cITP patients suffered from fatigue/asthenia. Transfusions were twice as frequent in cITP patients as in the overall ITP population, and mortality was also increased in the cITP population ( Table 2 ).

In addition, mean and median platelet counts at baseline were lower in cITP patients than in the overall ITP population and lower in children than in adults. At the end of follow-up, total ITP patients still had higher mean platelet count than cITP patients (126 vs. 122.4 × 10 ^9^ /L; Table 2 ).

### Prevalence and Incidence of Immune Thrombocytopenia in Spain

The prevalence of ITP in the general population was 10.8 (95% CI: 9.7–11.9) per 100,000 population. Children had a lower prevalence rate (7.5 [95% CI: 6.4–8.6]) compared to adults (11.7 [95% CI: 10.6–12.8]; *p* < 0.001). This trend was also observed for cITP. The prevalence rate was 2.7 (95% CI: 2.5–2.9), 1.7 (95% CI: 1.5–1.9), and 3.0 (95% CI: 2.8–3.2) for the whole ITP cohort, children, and adult patients, respectively ( Table 3 ).

**Table 3:** Incidence and prevalence of immune thrombocytopenia

Total		
Population on December 31, 2021	1,818,588	
Patients with a diagnosis of ITP	197	
New diagnoses of ITP	28	
ITP	197	
Prevalence rate (100,000 inhabitants)	10.8	(95% CI: 9.7–11.9)
Incidence rate (100,000 inhabitants)	1.5	(95% CI: 1.4–1.7)
cITP	50	–
Prevalence rate (100,000 inhabitants)	2.7	(95% CI: 2.5–2.9)
Incidence rate (100,000 inhabitants)	0.3	(95% CI: 0.2–0.4)
**Paediatric** (<18 years)		
Population on December 31, 2021	360,587	
Patients with a diagnosis of ITP	27	
New diagnoses of ITP	8	
ITP	27	
Prevalence rate (100,000 inhabitants)	7.5	(95% CI: 6.4–8.6)
Incidence rate (100,000 inhabitants)	2.2	(95% CI: 2.0–2.4)
cITP	6	
Prevalence rate (100 000 inhabitants)	1.7	(95% CI: 1.5–1.9)
Incidence rate (100 000 inhabitants)	0.5	(95% CI: 0.3–0.7)
**Adults** (≥18 years)		
Population on December 31, 2021	1,458,001	
Patients with a diagnosis of ITP	170	
New diagnoses of ITP	20	
ITP	170	
Prevalence rate (100,000 inhabitants)	11.7	(95% CI: 10.6–12.8)
Incidence rate (100,000 inhabitants)	1.4	(95% CI: 1.2–1.6)
cITP	44	–
Prevalence rate (100,000 inhabitants)	3.0	(95% CI: 2.8–3.2)
Incidence rate (100,000 inhabitants)	0.3	(95% CI: 0.2–0.4)

Abbreviations: CI, confidence interval; cITP, chronic immune thrombocytopenia; ITP, immune thrombocytopenia.

However, the annual incidence rate of ITP in adults (1.4 per 100,000 population [95% CI: 1.2–1.6]) was lower than in children (2.2 [95% CI: 2.0–2.4]; *p* < 0.001). The general incidence rate was 1.5 (95% CI: 1.4–1.7). Annual incidence for cITP patients was 0.3 per 100,000 people, and it was higher in children (0.5 per 100,000 people). Incidence and prevalence data are presented in Table 3 .

### Treatment Trends

Overall, the majority of diagnosed patients received treatment (98.0%; Table 4 ). Corticosteroids alone (69.0%) or associated with IVIG (13.7%) were widely used in the first-line setting (84.4% of treated patients) and were administered for a median of three cycles (P25–P75, 2–3). IVIG was used in 28.9% of patients in the first-line setting. Immunosuppressants were the most commonly used drugs in the second-line. There was a similar use of rituximab and eltrombopag, and romiplostim was prescribed less frequently than the other TPO-RAs. Fostamatinib was used in 4.5% of adult patients. Only three children required third-line treatment, and each used a different drug (romiplostim, immunosuppressants, or corticosteroids + immunomodulators), and only one had to restart the cycle with the combination of corticosteroids and IVIG. Adult patients who received third-line treatment were most likely to receive TPO-RA (32.4%), followed by immunosuppressants (29.4%) and corticosteroids + immunomodulatory drugs (20.6%). Those who required additional treatment were given corticosteroids (17.6%) or corticosteroids + IVIG (11.8%).

**Table 4:** Treatment/Medication prescribed to the population of study

	Total ITP patients			
Study groups	Paediatric (<18 years)	Adults (≥18 years)	Total	*p* -Value
**Number of patients**	27 (13.7%)	170 (86.3%)	197 (100%)	
**Therapeutic procedures**				
***Splenectomy, N*** **(%)**				
Before index date a	5 (18.5)	26 (15.3)	31 (15.7)	
After index date a	0 (0)	4 (2.4)	4 (2)	
During active period: pre- and postindex date a	5 (18.5)	30 (17.6)	35 (17.8)	0.912
2012	2 (7.4)	11 (6.5)	13 (6.6)	
2013	1 (3.7)	10 (5.9)	11 (5.6)	
2014	1 (3.7)	4 (2.4)	5 (2.5)	
2015	0 (0)	1 (0.6)	1 (0.5)	
2016	1 (3.7)	0 (0)	1 (0.5)	
2017	0 (0)	1 (0.6)	1 (0.5)	
2018	0 (0)	1 (0.6)	1 (0.5)	
2019	0 (0)	2 (1.2)	2 (1.0)	
**Treatments**				
**Patients in treatment,** ***N*** **(%)**				
No treatment	2 (7.4)	2 (1.2)	4 (2)	
With treatment	25 (92.6)	168 (98.8)	193 (98.0)	
***First line of treatment, N (%)***	**25**	**168**	**193**	0.097
Corticosteroids	15 (55.6)	121 (71.2)	136 (69.0)	
Immunoglobulin G (IVIG)	6 (22.2)	24 (14.1)	30 (15.2)	
Corticosteroids + IVIG	4 (14.8)	23 (13.5)	27 (13.7)	
* Not requiring further cycle treatment, N (%)*	6	30	36	
* Requiring****second cycle treatment*** , *N (%)*	**19**	**138**	**157**	0.424
Corticosteroids	10 (52.6)	76 (55.1)	86 (54.8)	
IVIG	0 (0)	0 (0)	0 (0)	
Corticosteroids + IVIG	9 (47.4)	62 (44.9)	71 (45.2)	
* Not requiring further cycle treatment, N (%)*	*1*	*20*	*21*	
* Requiring****third cycle treatment*** , *N (%)*	**18**	**118**	**136**	0.909
Corticosteroids	9 (50)	64 (54.2)	73 (53.7)	
IVIG	0 (0)	0 (0)	0 (0)	
Corticosteroids + IVIG	9 (50)	54 (45.8)	63 (46.3)	
* Not requiring further cycle treatment, N (%)*	15	117	132	
*And not requiring second-line treatment, N (%)*	*5*	*28*	*33*	
* Requiring****fourth cycle treatment*** , *N (%)*	**3**	**1**	**4**	**0.008**
Corticosteroids	0 (0)	0 (0)	0 (0)	
IVIG	0 (0)	0 (0)	0 (0)	
Corticosteroids + IVIG	3 (100)	1 (100)	4 (100)	
* Not requiring further cycle treatment, N (%)*	*3*	*1*	*4*	
*And not requiring second line of treatment, N (%)*	*1*	*1*	*2*	
***Second line of treatment, N (%)***	**12**	**89**	**101**	0.630
TPO-RA	3 (25.0)	19 (21.3)	22 (21.8)	
Romiplostin	0 (0)	8 (9)	8 (7.9)	
Eltrombopag	3 (25.0)	11 (12.4)	14 (13.9)	
Fostamatinib	0 (0)	4 (4.5)	4 (4.0)	
Rituximab	2 (16.7)	12 (13.5)	14 (13.9)	
Immunosuppressants b	7 (58.3)	45 (50.6)	52 (51.5)	
Corticosteroids + immunomodulators	0 (0)	9 (10.1)	9 (8.9)	
* Not requiring further treatment, N*	*9*	*55*	*64*	
***Third line of treatment, N (%)***	**3**	**34**	**37**	0.914
TPO-RA	1 (33.3)	11 (32.4)	12 (32.4)	
Romiplostin	1 (33.3)	7 (20.6)	8 (21.6)	
Eltrombopag	0 (0)	4 (11.8)	4 (10.8)	
Fostamatinib	0 (0)	3 (8.8)	3 (8.1)	
Rituximab	0 (0)	3 (8.8)	3 (8.1)	
Immunosuppressants	1 (33.3)	10 (29.4)	11 (29.7)	
Corticosteroids + immunomodulators	1 (33.3)	7 (20.6)	8 (21.6)	
* Restarting cycle treatment, N (%)*	1 (33.3)	10 (29.4)	11 (29.7)	
Corticosteroids	0 (0)	6 (17.6)	6 (16.2)	
IVIG	0 (0)	0 (0)	0 (0)	
Corticosteroids + IVIG	1 (33.3)	4 (11.8)	5 (13.5)	

Abbreviation: ITP, immune thrombocytopenia; IVIG, intravenous immunoglobulin; TPO-RA, thrombopoietin receptor agonist.

a Index date refers to the patient index date (each individual enters the study in a different moment)

b Danazol, cyclophosphamide, mycophenolate mofetil, azathioprine.

Between 2012 and 2019, 5 and 30 splenectomies/splenic embolisations were performed in children and adults, respectively, all in cITP patients ( Table 4 ).

### Health Care Resources and Management Costs Associated with Immune Thrombocytopenia

The use of health care resources, which included activities related to medical practice and complementary tests, and work disability are described in Table 5 . Visits to primary care, specialised care, and emergency departments resulted in an average of 13.9, 6.6, and 1.2 episodes per year in the total ITP population; 15, 9.7, and 1.4 times, respectively, in cITP patients. Regarding hospitalisation, 48.2% of ITP patients and 86.0% of cITP patients were hospitalised at least once a year during follow-up, with a mean (SD) of 0.8 (0.9) admissions per ITP patient and 1.5 (0.9) per cITP patient per year, and a mean (SD) of 4.9 (5.8) and 10.7 (5.1) days of stay, respectively. Significant differences were found between children and adults with ITP in day hospital visits, laboratory tests, and other tests. Children with ITP spent more time in the hospital than adults (mean [SD], 1.6 [1.0] vs. 1.1 [0.8] days), while adults with ITP had more laboratory tests (mean [SD], 7.8 [3.0] vs. 11.7 [3.5]) and other tests (mean [SD],1.2 [0.8] vs. 3.4 [1.0]). These differences were more pronounced in the cITP population, where children spent a mean (SD) of 12.5 (0.5) days in hospital compared to 1.3 (0.75) days for adults. In addition, children with cITP underwent a mean (SD) of 11.7 (2.1) laboratory tests and adults, 15.4 (2.1) per year. Finally, other tests were performed more often in adults than in children with cITP (mean [SD],1.5 [0.8] vs. 3.7 [0.8]). Regarding work incapacity, 26.6% of adult patients with ITP required sick leave and spent an average of 16.3 (SD, 37.8) days of sick leave annually.

**Table 5:** Resources use (average patient/year; annualized)

	Total ITP population				Chronic ITP population			
Study groups	Paediatric (<18 years)	Adults (≥18 years)	Total	*p* -Value	Paediatric (<18 years)	Adults (≥18 years)	Total	*p* -Value
Number of patients ( *n* , %)	27 (13.7%)	170 (86.3%)	197 (100%)		6 (12%)	44 (88%)	50 (100%)	
Medical practice a								
Primary care visits (mean, SD)	12 (8.1)	14.2 (7.9)	13.9 (7.9)	0.183	15 (8)	15 (4)	15 (8)	0.500
Specialized visits b (mean, SD)	6.2 (3.1)	6.6 (3.5)	6.6 (3.5)	0.569	11.2 (1.2)	9.5 (3.5)	9.7 (3.4)	0.120
Emergency rooms visits (mean, SD)	0.9 (1.0)	1.2 (1)	1.2 (1)	0.136	1.33 (1.03)	1.45 (0.73)	1.4 (0.8)	0.999
Day hospital sessions (mean, SD)	1.6 (1.0)	1.1 (0.8)	1.2 (0.9)	**0.006**	12.5 (0.5)	1.3 (0.75)	1.5 (0.8)	**0.001**
Hospitalized patients, ( *n* , %)	13 (48.2)	82 (48.2)	95 (48.2)	0.993	5 (83.3)	38 (86.4)	43 (86.0)	0.999
Average/patient hospital admissions (mean, SD)	0.7 (0.9)	0.8 (0.9)	0.8 (0.9)	0.552	1.2 (1)	1.6 (1)	1.5 (0.9)	0.300
Average/patient of days in hospital (mean, SD)	4.3 (5.6)	5 (5.8)	4.9 (5.8)	0.574	11 (6.5)	10.7 (5)	10.7 (5.1)	0.800
Complementary tests a								
Laboratory tests c (mean, SD)	7.8 (3)	11.7 (3.5)	11.2 (3.7)	**<0.001**	11.7 (2.1)	15.4 (2.1)	15 (2.4)	**0.002**
Conventional radiology (mean, SD)	0.6 (0.5)	0.7 (0.7)	0.7 (0.7)	0.509	1 (0)	1 (0)	1 (0)	0.999
Computed tomography (mean, SD)	0.8 (0.4)	0.9 (0.4)	0.9 (0.4)	0.063	0.8 (0.4)	0.9 (0.5)	0.9 (0.4)	0.500
Magnetic nuclear resonance (mean, SD)	1.9 (1)	1.9 (0.9)	1.9 (0.9)	0.995	3.5 (0.5)	3.1 (0.3)	3.1 (0.3)	**0.008**
Other tests d (mean, SD)	1.2 (0.8)	3.4 (1)	3.1 (1.2)	**<0.001**	1.5 (0.8)	3.7 (0.8)	3.4 (1.1)	**<0.001**
Work disability a								
Patients in sick leave ( *n* , %)	0	45 (26.6)	45 (22.9)	**0.002**	0	3 (6.8)	3 (6)	0.999
Average/patient sick leave, days (mean, SD)	0	16.3 (37.8)	14.1 (35.5)	**0.026**	0	2.6 (9.6)	2.3 (9.1)	0.999

Abbreviations: ITP, immune thrombocytopenia; SD, standard deviation.

Values expressed in percentage (%) or mean (SD). *p* -value denotes statistical significance.

a It includes costs related to ITP.

b Only in haematology and internal medicine departments.

c Any conventional lab analysis request.

d Lumbar puncture, bone marrow examination, and scintigraphy.

The distribution of patient costs is shown in Table 6 . Mean (SD) annual total cost for ITP patients was €10,741 (11,285). This cost was almost doubled for patients with cITP (€19,809 [14,656]). For patients with cITP, the mean annual cost of specialised care (€891 [€311]) was twice the cost of primary care (€357 [€175]). Medication costs were the most expensive item (€11,597 [€13,885]) followed by hospitalisation (€5,165 [€2,467]).

**Table 6:** Costs distribution (average patient/year; annualized; EUR)

	Total ITP population				Chronic ITP population			
Study groups	Paediatric (<18 years)	Adults (≥18 years)	Total	*p* -Value	Paediatric (<18 years)	Adults (≥18 years)	Total	*p* -Value
Number of patients	27 (13.7%)	170 (86.3%)	197 (100%)		6 (12%)	44 (88%)	50 (100%)	
*Medical practice*								
Primary care medical visit	278 (188)	329 (182)	322 (183)	0.183	344 (87)	359 (185)	357 (175)	0.500
Specialized care medical visit	572 (287)	610 (326)	605 (321)	0.569	1.027 (108)	872 (326)	891 (311)	0.120
Emergency medical visit	109 (113)	146 (120)	141 (120)	0.136	157 (121)	171 (86)	169 (89)	0.999
Day hospital sessions	295 (194)	202 (154)	215 (162)	**0.006**	462 (101)	248 (138)	274 (151)	**0.001**
Hospitalisation	2,084 (2,704)	2,410 (2,811)	2,365 (2,792)	0.574	5,290 (3,131)	5,148 (2,407)	5,165 (2,467)	0.800
*Complementary tests*								
Laboratory tests	173 (68)	261 (79)	249 (83)	**<0.001**	260 (48)	343 (47)	333 (54)	**0.002**
Conventional radiology	11 (9)	13 (13)	12 (13)	0.509	18.5 (0)	18.5 (0)	18.5 (0)	0.999
Computed tomography	75 (41)	89 (37)	87 (38)	0.063	80 (39)	94 (44)	92 (43)	0.500
Nuclear magnetic resonance	328 (182)	328 (158)	328 (161)	0.995	620 (97)	547 (51)	556 (62)	**0.008**
Other tests	44 (29)	127 (38)	116 (46)	**<0.001**	56 (31)	138 (31)	128 (41)	**<0.001**
Medication cost	4,246 (7,182)	4,977 (9,662)	4,876 (9,349)	0.707	12,378 (8741)	11,490 (14,516)	11,597 (13,885)	0.400
**Total health care cost**	8,215 (9,557)	9,492 (11,190)	9,317 (10,967)	0.575	20,693 (10,830)	19,428 (14,805)	19,580 (14,300)	0.700
**Indirect costs (sick leave days)**	0	1,650 (3823)	1,423 (3,595)	**0.026**	0 (0)	260 (974)	229 (917)	0.500
**Total cost**	8,215 (9,557)	11,142 (11,509)	10,741 (11,285)	0.211	20,693 (10,830)	19,688 (14,656)	19,809 (14,162)	0.700

Abbreviation: ITP, immune thrombocytopenia.

*p* -value denotes statistical significance.

Values expressed as mean (standard deviation) per patient/year; average/patient/year in EUR.

## Discussion

To the best of our knowledge, this is the first study to focus on the epidemiology, characteristics, treatment choices, health care resources and costs of the primary ITP population in children and adults in Spain. The information gathered here captures the reality of patients living with ITP and cITP between 2014 and 2020. Data show that patients with ITP have a unique set of characteristics, in terms of comorbidities, and use of health care resources, with all the strengths and limitations of the methodology used ( **Visual Summary** ).

Patients living with ITP experience a range of symptoms, such as the risk of bleeding or fatigue, which negatively affect their HRQoL. 1 2 6 7 17 24 In our study, epistaxis was the most common type of bleeding, followed by genitourinary and gastrointestinal bleeding. Major bleeding requiring hospital admission, was only 8.1% of cases in the ITP population (16.0% in cITP patients), despite many patients had risk factors for haemorrhages.

Epidemiological data in our analysis showed that the prevalence of ITP in Spain was 10.8 (95% CI: 9.7–11.9) per 100,000 inhabitants in 2021, and that of cITP, 2.7 (95% CI: 2.5–2.9), similar to results obtained in other locations. 12 25 In addition, adults had a higher prevalence of ITP and cITP than children, which has been previously reported in the literature. 12 26 27 The incidence was higher in children, similar to previous findings. 25

Regarding treatments, previous data from the Spanish ITP population was reported by Palau et al. who performed an observational study using information from the Haematology Services Registry between 2009 and 2011. 28 In their study, 81.1% of diagnosed patients received treatment, in contrast to our study, where 98.0% of diagnosed patients were treated. These frequencies are in contrast to other international studies reporting 50% and 75% of patients treated. 29 30 However, study populations were different in all cases, a situation that should be considered to properly analyse these data. For example, the selection periods differed in all studies compared to our study (we used data from 2014 to 2020): Palau et al. reported data from 2009 to 2011, 28 Weide et al. from 1995 to 2014, 29 and Depré et al. from 1996 to 2016. 30 In addition, our study was at a national level (BIG-PAC® has demonstrated representativeness at a national level), 21 while the study by Palau et al. was regional, in the one by Weide et al., patients were in a community-based oncology group practice in Germany and only outpatients treated by haematologists would be considered, 29 and Depré et al., patients were from a single institution. 30

In accordance with recommendations in guidelines, in our study corticosteroids were the most commonly used first-line treatment (in 82.7% of cases in our study; 73.4% in the study by Palau et al.), 28 and patients were reexposed to corticosteroids for a median of 3 cycles (in Palau et al. 59.5% of patients received corticosteroids for more than 6 weeks). Prolonged use of this type of therapy is known to have important side effects (hyperglycaemia, hypertension, mood and sleep changes, Cushing's syndrome, osteoporosis, etc.), 31 increasing disease burden, and contributing to decreasing patients' HRQoL. 14 32 It has also been shown to adversely affect growth and development in children. 31 Nevertheless, a recent review suggested that corticosteroids remain the main drug administered in current practice for ITP. 32 Romiplostim and eltrombopag were approved by the EMA in 2009 33 and 2010, 34 respectively. TPO-RAs have been available as second-line treatment in Spain since 2010 to 2011, initially as therapies to be considered when splenectomy was contraindicated, or patients were not willing to undergo the surgical procedure. 35 Currently, according to the American Society of Hematology 36 and the International Consensus Report, 11 these agents constitute a recommended second-line therapy immediately after corticosteroid failure. It is therefore striking to see that immunosuppressants and immunomodulatory drugs (danazol, cyclophosphamide, mycophenolate, and azathioprine) were the most commonly used second-line drugs in our study. In addition, it should be noted that, contrary to recommendation in guidelines, 70.5% of the patients in this study were treated with three or more cycles of corticosteroids (with or without IVIG) before initiating second-line therapy. This is a substantial gap from current guidelines, 11 36 which recommend a rapid switch to second-line therapy after a first course of steroids. Similarly, a study conducted in Spain that included patients diagnosed with ITP between 2011 and 2012 showed that more than one-third of patients who were re-treated due to failure or loss of response to prior steroid therapy were re-exposed to corticosteroids for two additional cycles. 37 Similar to current findings, the previous study also showed that TPO-RA were used immediately after failure of first-line therapy in only 25% of patients. 37

Immunosuppressants and immunomodulatory drugs were previously used as alternatives due to their lower cost and greater availability, but according to their data on their efficacy and safety, they are currently only recommended in patients who cannot tolerate or do not respond to other evidence-based second-line therapies. 38 The fact that this study includes patients who are followed up in non-specialist clinics and in primary care may be the reason for these data. Thus, it is possible that the out-of-hospital setting and also the management by physicians who are not necessarily experts in this disease explains an overuse of immunosuppressants and immunomodulators with respect to current recommendations.

Four patients received fostamatinib as second-line treatment; this drug was recently approved by the EMA in late 2019, 14 and in Spain in June 2020. It is likely that, together with the available treatments, fostamatinib, and other recently approved drugs such as avatrombopag will change the treatment landscape and improve patient's health and HRQoL in the near future.

In 2011, splenectomy was considered the standard second-line treatment for ITP. 35 Since the availability of the TPO-RA has changed this practice, we decided to analyse the number of splenectomies performed before and after 2016 to confirm an apparent change in patient management. Indeed, this change is reflected in the number of splenectomies performed before and after 2016 (5 vs. 0 in children and 26 vs. 4 in adult patients). In terms of patient differences, all splenectomies were performed in cITP patients, according to guidelines. 11

ITP patients sometimes display worse symptomatology and HRQoL than patients with other chronic diseases. 39 We cannot directly compare the distribution of costs with other diseases because of the different time period, but it is worth noting that our results indicate that the annualised mean (SD) cost of hospitalisation episodes in the total population of patients with ITP was €2,365 (€2,792) and in cITP was €5,165 (€2,467). Previous data indicate that hospitalisation due to cardiovascular disease had a cost of €2,146 (€4,947) in 2019 and decreased over time, 40 and hospital admissions for patients with type 2 diabetes mellitus (2DM) initiating treatment with glucagon-like peptide-1 receptor agonists also had lower costs (mean [SD] 588.3 [1,642.6], data calculated in 2020 based on 2017-unit prices) than cITP patients and the total ITP population. In this last case, comparison of our data and data gathered by Norrbacka et al. indicated that primary care and specialist visits, laboratory tests, CT, NMR, and medication costs were higher for ITP and cITP patients than for those with 2DM. In fact, two- and three-fold increases were observed in specialist visit, laboratory tests, NMR, and medication with regards to 2DM versus ITP, while three- to six-fold increases were evident in the same categories when compared to cITP costs. 41 ( Table 6 ).

This retrospective study has the inherent limitations intrinsic to these types of studies: some variables may be missing or there may be selection bias. In addition, as the BIG-PAC® database is an administrative database with information from seven different health care sectors, limitations inherent to its nature may be present (the data source may be incomplete, and disease categorisation, patient classification or cost range may be subject to bias). For example, if the primary ITP diagnosis were incorrect, and the specialists did not remove it from the medical history after confirming a wrong diagnosis, this patient would be considered to have an ITP diagnosis, which would overestimate our population. Lastly, it is worth noting that, although hospitalisations were associated with ITP, they could be caused by reasons other than bleeding, but still attributable to ITP according to the specialist. Future studies including the cause of admission could help to identify the impact of treatments, for example, negative outcomes produced by infections caused by the treatment, or, on the contrary, early treatment improving patient's HRQoL.

## Conclusion

This is the first study to analyse aspects of the epidemiology, treatment landscape, health care resources use, and management costs of ITP in Spain. Paediatric and adult patients with ITP suffer a significant burden, which is accentuated by the chronification of the disease. In addition, the choice of treatment, which highlights an overuse of corticosteroids coupled with an underuse of two-line treatments such as TPO-RA, increased medical visits, and sick leave, among others, contribute to the increase in direct and indirect costs, which seem to be higher than in other chronic pathologies. Therefore, information on the above variables could be of interest to health authorities and health policy-makers.

## Notes

**What is Known about This Topic?**

- Patients with ITP are prone to bleeding, fatigue, and poor HRQoL.
- In Europe, the prevalence and annual incidence of ITP is approximately 10 to 50 per 100,000 and 1 in 25,600 to 37,000 people, respectively.
- The disease behaves differently in children and adults.

**What does This Paper Add?**

- This is the first study of this kind to be conducted on the ITP population in Spain illustrating the situation of these patients in terms of different variables.
- The analysis of the records of a total of 1,818,588 patients reveals the true incidence and prevalence of the disease in Spain, baseline and clinical characteristics, treatment trends, use of health care resources, and management costs for paediatric and adult patients.
