# Supplementary material for: Epidemiology, Treatment Patterns, and Cost Analysis of Immune Thrombocytopenia in Spain between 2014 and 2020: A Population-based Study
Source: TH Open. 2024 Jul 8;8(3):e252–65. doi: 10.1055/a-2336-1062 (PMC11230702; doi:10.1055/a-2336-1062)
Supplement: Supplementary file 1 — Supplementary Material [file 10-1055-a-2336-1062-s24020007.pdf]

Supplementary Material

Supplementary Table S1 Inclusion and exclusion criteria

Inclusion criteria	Exclusion criteria
a) Patients with a diagnosis of ITP<br>b) Patients included in the database for ≥12 months, prior to study entry<br>c) Enrolled in the program for obtaining prescriptions (with recorded daily dose, time interval, and duration of each treatment administered; ≥2 prescriptions during the follow-up period)<br>d) Regular monitoring of patients (≥2 health records in the computer system)	a) Subjects transferred to other centers, displaced, or out-of-area<br>b) Residents of nursing homes<br>c) Patients with secondary immune thrombocytopenia or any other cause of thrombocytopenia other than primary ITP

Abbreviation: ITP, immune thrombocytopenia.

Supplementary Table S2 International Classification of Diseases, 9th Edition, Clinical Modification codes of sign and symptoms of immune thrombocytopenia

	ICD-9-CM codes
**Bleeding episodes**	
Intracranial bleeding	430, 431, 432.x, 852.x, 853.x
Gastrointestinal bleeding	456.0, 456.20, 459.0x, 530.21, 530.7, 530.82, 531.0x, 531.2x, 531.4x, 531.6x, 532.0x, 532.2x, 532.4x, 532.6x, 533.0x, 533.2x, 533.4x, 533.6x, 534.0x, 534.2x, 534.4x, 534.6x, 535.01, 535.11, 535.21, 535.31, 535.41, 535.51, 535.61, 535.71, 537.83, 537.84, 562.02, 562.03, 562.12, 562.13, 568.81, 569.3, 569.85, 578.x
Genitourinary bleeding	593.81, 596.7x, 596,89, 599.7x, 602.1x, 620.1, 621.4, 622.8, 626.2, 626.5, 626.7, 626.8, 626.9
Nasal bleeding (epistaxis)	784.7
Other events	285.1, 360.43, 362.43, 362.81, 363.61, 363.62, 363.72, 364.41, 372.72, 374.81, 376.32, 377.42, 379.23, 423.0x, 719.1x, 782.7, 784.8, 786.3x, 958.2, 997.02, 998.11, 99.04
**Fatigue/Asthenia**	780.79
**Transfusions**	99.0

Abbreviation: ICD-9-CM, International Classification of Diseases, Ninth Revision, Clinical Modification.

Supplementary Table S3 Anatomical Therapeutic Chemical Classification System codes for the treatment drugs and ICD-9-CM codes for splenectomies[a]

Drug	Code
Azathioprine	L04AX01
Cyclophosphamide	L01AA01
Cyclosporin	L04AD01
Danazol	G03XA01
Dexamethasone	H02AB02
Eltrombopag	B02BX05
Fostamatinib	B02BX09
Intravenous immunoglobin therapy	J06BA
Methylprednisolone	H02AB04
Mycophenolate	L04AA06
Prednisolone	H02AB06
Prednisone	H02AB07
Rituximab	L01XC02
Romiplostim	B02BX04
Partial and total splenectomies	41.43 and 41.5

Abbreviation: ICD-9-CM, International Classification of Diseases, Ninth Revision, Clinical Modification.

[a]World Health Organization. The Anatomical Therapeutic Chemical Classification System with Defined Daily Doses (ATC/DDD). Accessed June 7, 2024 at: https://www.who.int/standards/classifications/other-classifications/the-anatomical-therapeutic-chemical-classification-system-with-defined-daily-doses.

 © 2024. The Author(s).

Supplementary Table S4 Table of costs (unit cost [€])[a]

**Medical visits**	
Primary care medical visit	23.19
Specialized care medical visit[b]	92.00
Emergency medical visit	117.53
Hospitalization (1 day)	480.90
Day hospital visit	185.00
**Complementary tests**	
Laboratory tests	22.30
Conventional radiology	18.50
Computed tomography	96.00
Magnetic nuclear resonance	177.00
Other tests	37.50
**Pharmaceutical prescription**	Retail price
**Work disability—indirect costs**	
Cost per day not worked[c]	101.20

[a]It includes costs related to immune thrombocytopenia.
[b]Only in haematology and internal medicine departments.
[c]National Institute on Statistics. Average employment earnings by age and sex. (Adapted from Sicras-Mainar et al [2019][21] and Instituto Nacional de Estadística[23].)

 © 2024. The Author(s).
